# Incidence of Horner syndrome associated with neuroblastic disease

**DOI:** 10.1007/s00381-020-04966-z

**Published:** 2020-11-10

**Authors:** Katarzyna Kuchalska, Monika Barełkowska, Katarzyna Derwich, Katarzyna Jończyk-Potoczna, Anna Gotz-Więckowska

**Affiliations:** 1 Karol Marcinkowski University of Medical Sciences, Poznań, Poland; 2 Department of Oncology, Hematology and Transplantology, Karol Marcinkowski University of Medical Sciences, Poznań, Poland; 3 Department of Paediatric Radiology, Poznań University of Medical Sciences, Poznań, Poland; 4 Department of Ophthalmology, Karol Marcinkowski University of Medical Sciences, Poznań, Poland

**Keywords:** Neuroblastoma, Ganglioneuroblastoma, Miosis, Ptosis

## Abstract

**Purpose:**

Horner syndrome (HS) manifests in unilateral ptosis, miosis, enophthalmos, and anhedonia. It is most commonly caused by trauma or surgical procedures, but can also occur in pediatric patients as a result of tumors, especially neuroblastoma (NBL). The objective of this study was to analyze the incidence of HS in patients diagnosed with NBL.

**Methods:**

A retrospective analysis of data collected at the Department of Pediatric Oncology, Hematology, and Transplantology from 2004 to 2019 was performed. The study group included 119 patients younger than 18 years old, with 62 girls and 57 boys. All of them were diagnosed with a neuroblastic tumor.

**Results:**

Among the 119 patients, eight children (6.72%) were diagnosed with HS associated with NBL. Three of these patients presented to the clinic with HS, whereas HS developed after the surgical procedure to remove the tumor in four patients. The adrenal gland was the most frequent localization of the tumor. However, HS occurred more frequently in patients with mediastinum tumors. As a presenting symptom, HS occurred in 2 of 11 cases (18.18%) with mediastinum localization. All of the patients with HS were younger than 2 years old.

**Conclusion:**

Investigation of the cause of isolated HS is crucial because it can be the first symptom of NBL. However, the surgical procedure itself increases the risk of HS as a complication of NBL treatment.

## Introduction

**Fig. 1:**
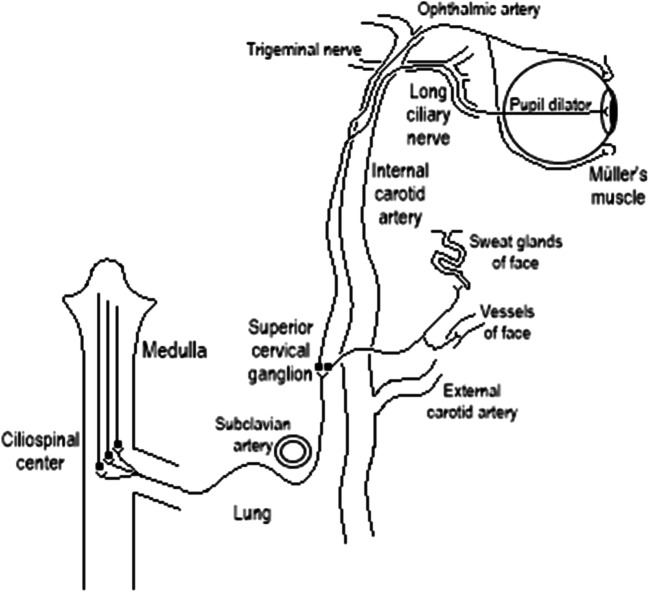
Oculosympathetic tract

Horner syndrome (HS) manifests in unilateral miosis, ptosis, enophthalmos, and anhidrosis. In general, it is caused by an interruption in the oculosympathetic tract (Fig. 1) [1]. Horner syndrome most frequently occurs in the first year of life and is often caused by trauma (65%). Congenital HS is more common than acquired HS (55% vs. 45%) [2].

Neuroblastoma (NBL) is a small blue round cell tumor in histopathology. These tumors most commonly occur in the first 12 months of life and are localized in the abdomen, especially the adrenal medulla. The International Staging System defines four stages within the 1, 2, and 4S for patients with localized tumors, which should be operated firstly. These patients have a good prognosis. In the group of patients with stage 4S NBL, children are younger than 12 months old, and metastases are generally limited to the liver, skin, and bone marrow, which may disappear after removal of the first NBL focus. The other ophthalmologic symptoms of NBL include proptosis, strabismus, ecchymosis, exophthalmos, palpable supraorbital masses, edema of the lids, conjunctiva, papilledema, dilated retinal veins, paresis of the external rectus muscle, optic atrophy, and retinal hemorrhage [3, 4]. The purpose of the study was to analyze HS occurrence in patients diagnosed with a neuroblastic tumor.

## Methods

Data were collected at the Department of Pediatric Oncology, Hematology, and Transplantology from 2004 to 2019. The inclusion criteria were patients aged under 18 years on the day of admission and confirmation of the neuroblastic disease diagnosis. A total of 119 children were included. Between them, there were 108 NBLs, 9 ganglioneuroblastomas, and 2 ganlioneuromas. There were 62 girls (52%) and 57 boys with an average age of 2.2 years. However, 59% of the group were younger than 2 years old (70 patients). The adrenal gland was the most frequent localization of the tumor.

## Results

Eight cases of HS were found in the whole group (6.72%), representing an incidence of 7.41% among patients with NBL. Three of them had HS as the presenting symptom (2.78%), while one patient had isolated HS as the only manifestation of NBL (0.93%). In this group, there was one patient with congenital HS (0.93%). As a presenting symptom, HS occurred in 2 of 11 patients with tumors localized in the mediastinum (18.18%). Another four patients were diagnosed with HS after surgery (3.70%), meaning that ptosis or miosis was a complication of the surgical procedure. All of the patients with HS were younger than 2 years old. The results are shown in Table 1.

**Table 1:** Occurrence of Horner syndrome (HS) in the group of pediatric patients with neuroblastoma (NBL; 108 patients) and in the group of patients with all neuroblastic tumors (119 patients)

	No. of patients	Incidence in the NBL group	Incidence in the whole group
HS as a manifestation	3	2.78%	2.52%
Post-procedure HS	4	3.70%	3.36%
HS occurred during hospitalization (not post-procedure)	1	0.93%	0.84%

**Fig. 2:**
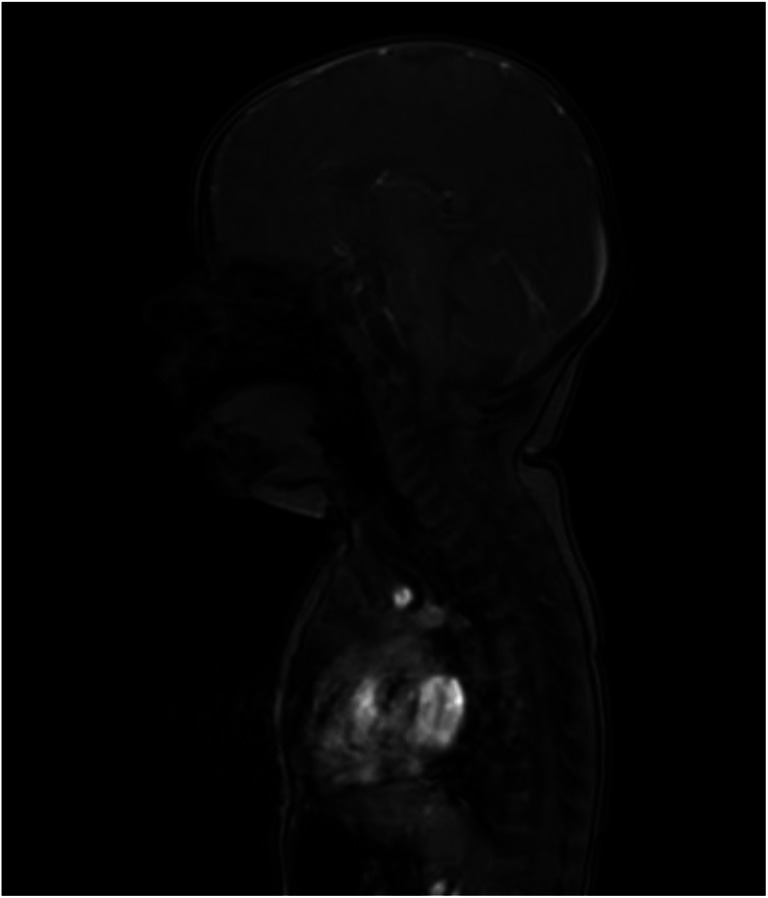
MRI of patient with a tumor adjacent to left subclavian artery

The first patient was a 3-month-old girl who presented with left-sided ptosis and miosis. She did not suffer from any other symptoms. In the MRI, a tumor adjacent to the left subclavian artery was found (Fig. 2). A biopsy confirmed the suspicion of NBL, and the tumor was classified as stage 2. The patient was treated by chemotherapy (CHT) with vincristine and surgery. She achieved remission; however, the symptoms of HS were still present.

**Fig. 3:**
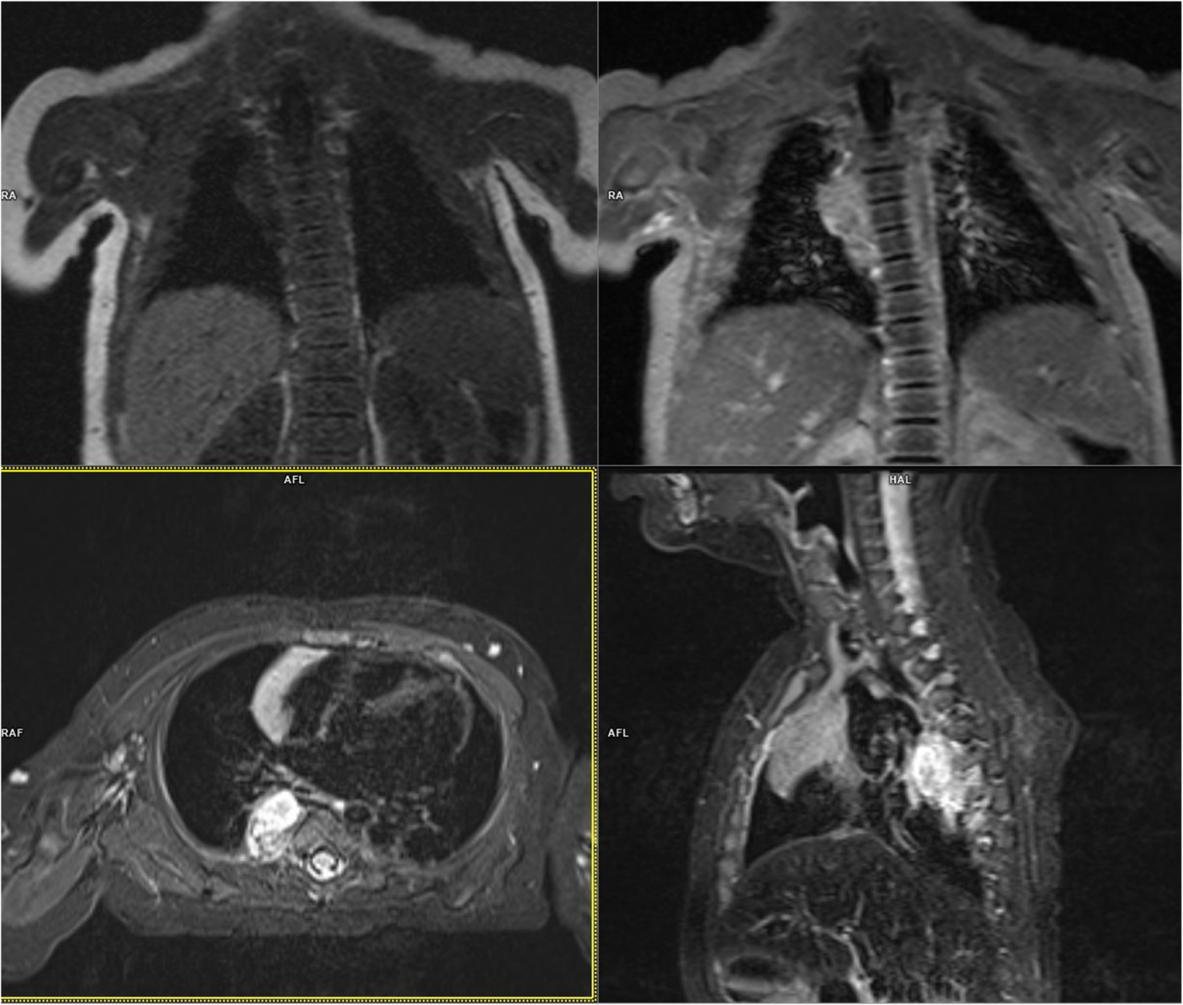
MRI of a boy with a tumor localized in back mediastinum

A boy with congenital right-sided HS had subcutaneous nodules and palpable tumors in the left side of the abdomen in addition to palpebral fissure narrowing. On imaging examination, the mass was found to have penetrated the intervertebral space (Fig. 3). Metastases in the liver, subcutaneous tissue, and scrotum were detected; thus the disease was classified as stage 4S. Chemotherapy was performed, resulting in a decrease in HS symptoms and a reduced number of subcutaneous nodules. The child finally achieved remission, but ptosis and anisocoria were still present.

**Fig. 4:**
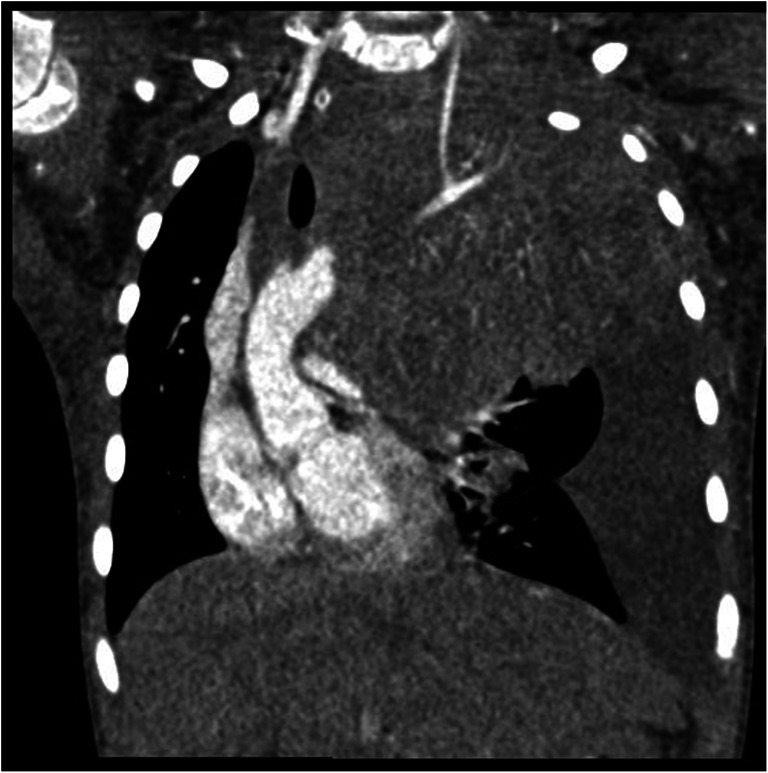
CT of patient with a NBL tumor involving aorta, heart, trachea and left bronchi

The next patient was admitted at 2 years of age. The boy had left-sided ptosis, hoarseness, vasodilation of the thoracic skin, and decreased warming of the lower extremities. Following the CT and biopsy, he was diagnosed with stage 4 NBL that was localized in the thoracic cavity (Fig. 4). Additionally, supraclavicular and cervical lymphadenopathy were detected. Tracheostomy, pleurectomy, and adjuvant chemotherapy were performed. The patient was then transferred to the surgery department.

Another patient with right-sided HS was admitted at 3 months of age after subtotal tumor removal surgery. The NBL in the thoracic cavity was classified as stage 4. After eight cycles of CHT, the boy underwent a second surgery. Three months later, HS was still present.

A boy younger than 1 year old was diagnosed with NBL in stage 4. The symptoms of HS were observed after NBL removal. The tumor was localized in the thoracic cavity. Unfortunately, the child died.

Another patient in the post-procedure HS group presented with periodic apnea and cyanosis at 10 weeks of age, in addition to subcutaneous nodules on the lower extremities, torticollis, and recurring infections of the upper respiratory tract. A tumor in the neck was detected, along with numerous foci in the liver. He was diagnosed with stage 4S NBL and started treatment with one cycle of chemotherapy followed by surgery. After the procedure, he started to have problems with sucking reflex, dysphagia, right-sided HS, and increased pulmonary inflammation, so he spent 7 days in the ICU. Remission was achieved. Nevertheless, HS was still present.

The next patient was a 10-month-old girl with paravertebral NBL in the first stage. On admission, she had Harlequin syndrome and was in good condition. She started chemotherapy and surgery was planned. After the surgery, right-sided HS occurred. She achieved remission of neoplastic disease; however, anisocoria was still present at 2 years of age.

The last patient was born with respiratory problems, portal vein thrombosis, and a tumor in the mediastinum. He was diagnosed with NBL in stage 4. On his 13th day of life, the patient underwent subtotal removal surgery. After the operation, the patient’s condition was poor. One of the complications was right-sided HS. After a few cycles of chemotherapy, the child unfortunately died.

## Discussion

A study by Graef et al. also measured the occurrence of HS in children diagnosed with NBL. In this previous study, there were 24 cases of HS in a group of 524 children with NBL, representing an HS incidence rate of 4.60%, which is a smaller than that observed in our analysis (7.41%). HS was a presenting symptom in 1.30% of patients [4]. These findings can be compared with our result (2.78%) and the results of other researchers, with 2.22% (405 pediatric patients) reported by Musarella et al. and 13% (30 children) by Jaffe et al. [4, 5]. Based on these publications, the incidence of HS as a complication of NBL treatment can be compared. This incidence was 3.70% in our study, 2.10% in the group of 523 NBL cases, and 13% in 30 patients, where all of the tumors were localized in the mediastinum [4, 6]. According to research by Graef et al., development of HS during the course of NBL is associated with a better prognosis [6].

**Fig. 5:**
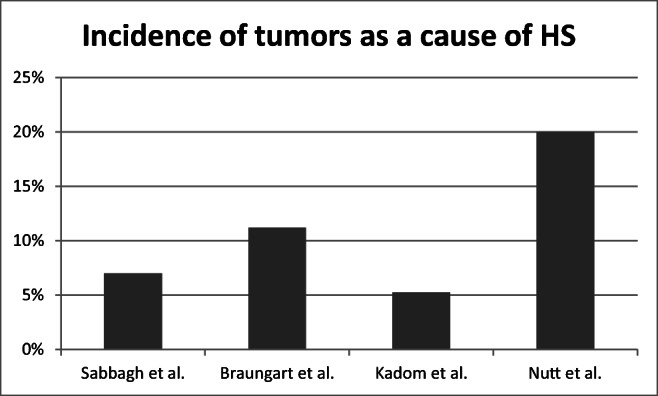
The results of different studies analyzing the causes of Horner syndrome (HS) [7–10]

An interesting finding is the incidence of specific causes in patients with isolated HS. The complexity of such analyses is demonstrated by the variety of results obtained in individual studies (Fig. 5). Nutt et al. described 10 different children suffering from HS, with two cases of NBL among them. Post-procedure symptoms occurred in another two patients [7]. In an extensive retrospective study by Sabbagh et al., the occurrence of tumors differed between pharmacologically confirmed cases and unconfirmed cases (7% vs. 18%), which was a similar finding to the postoperative HS group (21% vs. 36%). A total of 318 patients ranging from 4 to 87 years of age were included in this research, and the apraclonidine test was the deciding factor for a patient to qualify for the pharmacologically confirmed group [8]. Another interesting analysis of 38 CT and MRI scans showed tumor occurrence in 5% of pediatric patients with HS (two neoplasms; one NBL and one astrocytoma), whereas HS was associated with a procedure in only one patient (3%) [9]. According to a review of eight papers with 152 patients across a broad age range and with different diagnostic criteria, tumors caused HS in 11.18% of cases and NBL in 7.89% [10].

Ptosis in HS should not affect the development of vision. However, because of the cosmetic defect, it is important to note that the HS symptoms did not disappear during or even after the treatment in all of the presented cases. Nutt et al. described ptosis improvement in a patient with miosis 1 year following the NBL diagnosis. In the same paper, there was a description of atypical HS as a manifestation of NBL, which was recurring [7]. In the cases of HS caused by other problems, symptoms also did not disappear. Smith et al. described 20 pediatric patients with HS, finding only two improvements in postoperative cases and two remissions in HS induced by trauma [2].

In conclusion, investigation of the cause of HS is crucial and requires vigilance and appropriate imaging, as it can be the first or isolated manifestation of neuroblastoma. Moreover, the surgical procedures involved in the tumor treatment process significantly increase the incidence of ptosis and miosis.

## Data Availability

Data that support the findings of this study are available from the corresponding author, KK, upon reasonable request.
